# Measuring health professionals’ perceptions of communication contributing to medication incidents in hospitals - scale development and primary results of weekly perceived communication challenges

**DOI:** 10.1186/s12912-023-01455-x

**Published:** 2023-08-25

**Authors:** Tiina Syyrilä, Katri Vehviläinen-Julkunen, Santtu Mikkonen, Marja Härkänen

**Affiliations:** 1 Department of Nursing Science, Faculty of Health Sciences, University of Eastern Finland (UEF), PO Box 1627, Kuopio, 70211 Finland; 2 Kuopio University Hospital (KUH), Kuopio, Finland; 3 Department of Environmental and Biological Sciences, University of Eastern Finland (UEF), Kuopio, Finland; 4 Department of Technical Physics, Faculty of Science, Forestry and Technology, University of Eastern Finland (UEF), Kuopio, Finland

**Keywords:** Medication, Incident, Communication, Hospitals, Scale, Medication safety

## Abstract

**Background:**

Communication challenges are one of the main contributors for medication incidents in hospitals, but health professionals’ perceptions about variety of the contributing communication factors and the factors’ occurrence frequencies are studied little. This cross-sectional descriptive study aimed to (1) operationalize a literature-based framework into a scale for measuring health professionals’ perceptions of communication factors, which contribute to medication incidents either directly or indirectly in hospitals, (2) to measure the construct validity and internal consistency of the scale and (3) to describe the primary results of the measured weekly perceived communication challenges.

**Methods:**

The structured online questionnaire with 82 communication related items was developed based on a framework in literature. A content validity index of expert panelists’ answers was used for item reduction. Data was collected between November 1st, 2019, and January 31st, 2020, by convenience sampling. The study sample (n = 303) included multiple health professional groups in diverse specialties, unit types and organizational levels in two specialized university hospital districts in Finland. Exploratory factor analysis with Maximum Likelihood method and Oblique rotation produced a six factors scale consisting of 57 items and having acceptable construct validity and internal consistency.

**Results:**

The six communication factors contributing to medication incidents concerned (1) medication prescriptions, (2) guidelines and reporting, (3) patient and family, (4) guideline implementation,5) competencies and responsibilities, and 6) attitude and atmosphere. The most frequently perceived communication challenges belonged to the Medication prescription related factor. Detailed item frequencies suggested that the most usual weekly challenges were: (1) lack or unclarity of communication about medication prescriptions, (2) missing the prescriptions which were written outside of the regular physician-ward-rounds and (3) digital software restricting information transfer.

**Conclusions:**

The scale can be used for determining the most frequent detailed communication challenges. Confirmatory factor analysis of the scale is needed with a new sample for the scale validation. The weekly perceived communication challenges suggest that interventions are needed to standardize prescribing documentation and to strengthen communication about prescriptions given outside of regular ward-rounds.

**Supplementary Information:**

The online version contains supplementary material available at 10.1186/s12912-023-01455-x.

## Background

Annual global costs due to medication incidents are rising towards fifty billion US dollars and patients are caused harm or risk of harm in healthcare [1]. Global “Medication Without Harm” program published by WHO in 2017 [2] urged to halve medication incidents. Communication factors are found to be recurring main contributors to medication incidents [3–5]. Medication communication has been studied regarding specific communication challenges and interactions between patients and health professionals [5–8]. In literature technological communication solutions in medication processes have been described to enhance medication safety [7, 9, 10]. However, perceived frequencies of diverse communication challenges by professionals are still studied little. This needs more attention to direct interventions to the most optimal factors. Also, an overview of the primary communication challenges and communication promoting factors regarding medication safety in hospitals is needed [5].

Medication communication is affected by multifaceted issues. Researchers have defined medication communication as verbal, written, nonverbal or digital communication regarding medication between professionals, patients and family members and students in healthcare [3, 11]. Medication related communication concept in this study is understood wider than just as communication at the point-of-medication-administration. Hence, within the communication concept is included also communication in other medication related process phases than just in medication administration phase. Communication on diverse hierarchy levels is taken into account when the outcome affects medication care, even indirectly. For example, administrative communication about expectations to follow medication safety guidelines and rules in the organization may contribute to medication incidents as outcome. Similarly, lack of administrative communication regarding excessive workload during medication administration or reconciliation situations may contribute indirectly to medication incidents. Lack of communication about needed and available equipment or materials may have an impact on medication safety. For example, the lack of communication about the current stock level of necessary equipment or ordering process and ordering responsibilities may affect whether there are filter needles available or not when needed. It is recognized that there are also other factors than communication factors leading to lack of equipment. Nevertheless, in this study it is assumed, that if the equipment is missing, while it should be there, probably, some communication have not been optimal if substitute arrangements were not in place. Indirect aspects are included in the communication concept that communication challenges regarding both sharp end and blunt end contexts are covered. Sharp end meaning front line point of care, and blunt end meaning circumstances which are not in direct control of front line professionals.

According to literature, medication communication is affected by environmental, circumstance and staffing factors [5, 12–14]. Interprofessional hierarchical structure and diverse expectations about communication are described affecting medication safety [3, 15]. Several technical innovations have been deployed for strengthening medication communication. E-messaging between care providers [15] and artificial intelligence in combining prescription data with information provided by patients in electronic health records [16] have been investigated. Communication training strategies for health professionals have been trialled for improving medication communication; video recording has been used to increase reflectivity between clinicians when communicating about medication [17]. Simulation-based learning methods have been tested to observe clinicians’ awareness of erroneous prescriptions and professionals’ courage to report errors [18].

Patient safety incident reporting systems are used in many countries for collecting information about medication incidents. Communication factors contributing to medication incidents can usually be reported in them on a general level. For detailed information of communication factors is needed additional measurements. However, existing studies have focused on measuring specific challenges or testing technical solutions related to medication communication. The studies have evaluated communication in general terms as part of patient safety culture [19] and conducted satisfaction surveys for patients [13] or hospital consumer assessments [20]. Thus, choosing optimal interventions to enhance communication involves confronting a wide variety of communication factors and interventions. No scale yet exists for assessing professionals’ perceptions of communication factors contributing to medication incidents. Neither exist scales to measure relative frequency of diverse communication factors contributing to medication incidents. A new scale is needed for measuring type variety and frequency of communication challenges to prioritize which medication communication factors need actions most urgently. Such a scale is developed in this study.

To start the scale development for the study, a concept description was required. According to literature, a concept analysis of medication communication was conducted by Manias in 2010 [21]. She also concluded that a clinical tool is needed to assess medication communication. Later, Syyrilä with colleagues [22] developed a literature-based conceptual framework of medication incident related communication (Medication Incidents and Communication in Hospitals [MIComHos]), consisting of 128 operationalizable items. They used the framework to identify communication issues in medication incident reports to find evidence of the framework’s clinical relevancy. They suggested further development of the framework into a clinical scale [22] and further developed a concept of communication related to medication incidents [11]. Based on the concept analysis, the communication items relating to medication incidents can be grouped in to the six main categories: (1) communication dyads, (2) prescription related issues, (3) individual issues, (4) institutional/administrative issues, (5) contextual and process issues and (6) qualitative characteristics of communication [11].

## Methods

### Aims

This cross-sectional descriptive study aimed to (1) operationalize the MIComHos framework into a preliminary MIComHos-S1 (S1 = scale, version one) for measuring health professionals’ perceptions of communication factors, which contribute to medication incidents either directly or indirectly in hospitals, (2) to measure the construct validity and internal consistency of the scale and (3) to describe the primary weekly perceived communication challenges based on the survey data collected from health professionals for the scale development.

### Study design

The study design was cross-sectional and descriptive.

### Participants and data collection

#### Sampling

To generalize the descriptive results indicatively, the sample size was based on the total number of targeted healthcare professionals [23] in hospitals in Finland (93,000 healthcare professionals) and in the participating specialized healthcare organizations in the current study (20,000 healthcare professionals). A Raosoft [24] sample size calculator was used for calculating minimum sample size based on the studied population. According to Raosoft calculation, minimum of 377 respondents would allow to generalize the results in the population by error margin at 5% and confidence interval (CI) at 95%. Convenience sampling was used for securing variety in the sample [25]. Based on the current literature, the response rate was expected to be around 10% [26]. This means that to achieve the required minimum number (n = 377) of responses, the questionnaire was needed to send to minimum of 3770 health professionals (100% x 377)/ 10% = 3770). To secure achieving this minimum number of respondents in case the response rate would be even below 10%, it was decided to send the questionnaire up to 4000 professionals. However, one large clinic preferred not to participate to the survey, thus factually the survey was managed to send only to n = 3,892 healthcare professionals.

#### Inclusion and exclusion criteria of participants and settings

Inclusion criteria for choosing participating units or clinics for the digital survey were that the unit or wider clinical area provided medication care for somatic adult patients. The final 101 participating units included the following types: inpatient departments, outpatient clinics, intensive care units, operating theatres, recovery departments, day surgery units, emergency units and ambulance services. The targeted professionals for the digital survey respondents in included units or clinics were from all organizational levels, including nurses, physicians, pharmacists, clinical teachers in nursing and medicine, clinical specialists, patient safety specialists, managers, and chiefs.

Exclusion criteria for choosing the participating units and wider clinical areas were: The setting was psychiatric or paediatric. It was assumed that in these settings might have additional specific communication issues regarding the patients cared for [13].

#### Respondent recruitment

One nursing director was agreed to serve as contact person in both study organizations for facilitating implementation of the survey. The contact people gave contact details of the nurse- and medical directors and managers in the clinics. A study introduction was provided in person for directors, managers and specialists in all participating areas and PowerPoint slides were shared and asked to forward them to professionals in clinical areas before sending the survey link. The survey link was sent via email through chiefs, managers, or secretaries according to preference of each clinic or unit. Two reminders and additional cake drawing leaflets were sent via email and in person. Each unit was visited, and chocolate provided in person to remind about the survey in the middle of data collection.

### Study instrument

#### Framework-item’s reduction and questionnaire development

**Fig. 1:**
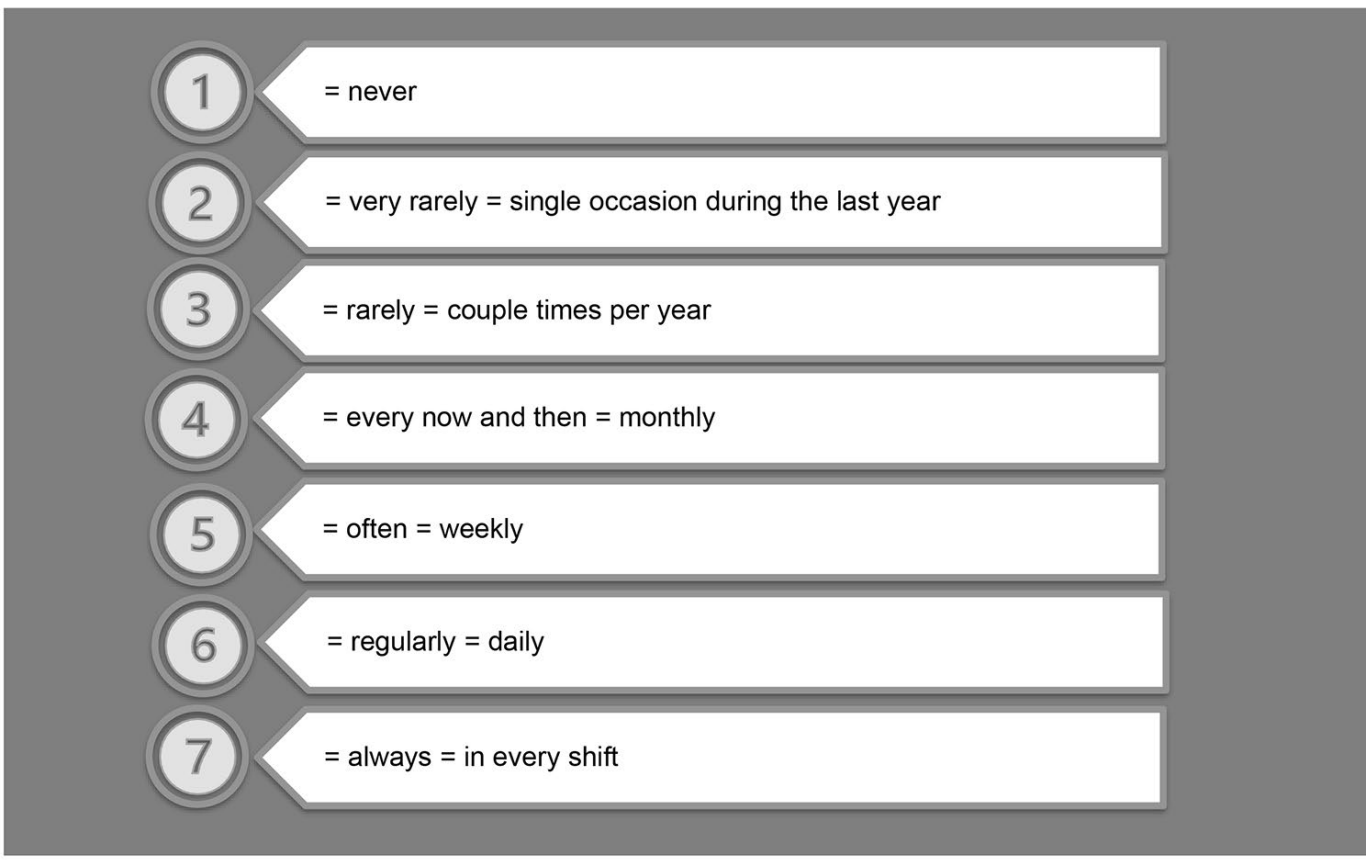
Seven-step Likert scale for measuring the perceived frequency of communication challenges (= items of factors)

Expert panel (n = 14) of health professionals and patient representatives, and content validity index [CVI] method were used for item reduction and amendments of the MIComHos framework [22] for the questionnaire. Forty-nine items were removed from the original framework based on the CVI of expert panelists’ answers. The CVI values of the retained items (79 items) were 0.88 for importance and 0.79 for clarity. Three additional questions were incorporated into the questionnaire based on the authors’ practical experience in hospitals. Two of them were background variables, and one item concerned look-alike–sound-alike characteristics of medication. The background variables of sex (CVR, 0.36) and age (CVR, 0.50) were discarded based on the low content validity scores. The final questionnaire consisted of 12 background variables and 82 communication items (94 total items). A seven-step Likert scale (Fig. 1) was set for measuring how often health professionals come across the detailed communication challenges (= items). The Eduix [27] software was used for building the digital questionnaire form and to collect the digital questionnaire data from respondents. The digital form was technically pilot tested by health professionals (n = 5). Minor technical and wording amendments were done after testing. Health professionals’ empirical perceptions of communication challenge frequencies were obtained using the structured digital survey in Finnish language. The survey items were translated into English for the publication and checked by the author team. An official translator was used to finalize the translation.

### Data collection

The digital questionnaire link and two reminders were sent via email to n = 3,892 healthcare professionals in two university hospital districts in Finland between November 1, 2019, and January 31, 2020.

### Data analysis

The IBM Statistical Package for the Social Sciences statistics for Windows (SPSS), Version 25.0 (Chicago, IL, USA) was used for statistical analysis of the data. Data for the background variables and Likert scale values was described using frequencies and percentages. The Likert scale values were used also for calculating factor levels as arithmetic mean values. The differences in factor levels were measured statistically between clinical unit types, hierarchy positions, “work experience length” and “if ward pharmacist was available or not” categories. These results with statistical significance values are described in a Supplementary Table 1.

#### Data preparation for the analysis

Missing values were checked to decide if multiple imputation (MI) was needed [28]. The MI method was used to replace missing data with neutral place holders to maximize the usage of the collected data, doing so without changing the data outcomes. Without MI, around half of the data would have been discarded due to listwise deletion in SPSS program, which would have been waste of valuable data and unethical action regarding effort from the respondents. Several preparation steps regarding missing data were required as standard process steps of MI procedure.

Missing values in the returned forms (n = 344) were checked, disqualifying n = 41 forms due to missing responses to over 50% of the questions, which was set as the cut-off point for MI [29]. To increase reliability, the variables with missing data ≥ 11% were removed [29, 30] from imputation because SPSS recommends a maximum of 10% of missing data per variable for imputation. After conducting variable removal in the remaining data, 4.98% was missing. Using Missing Completely at Random test (MCAR) was confirmed that the “missingness” was non-significant, allowing imputation (chi-square = 12431.670; Degrees of freedom (DF) = 12,206; Significance level (Sig.) = 0.075) [28]. The data was imputed using multiple MIs through 10 imputations, applying the Custom Fully Conditional Specification method and the Predictive Mean Matching (PMM) method, repeating for 200 iterations [28]. The imputed results were aggregated into a separate file for the final analysis of the data. After imputation, the imputed data (n = 303) and original data (n = 133 left after listwise deletion) were compared using independent samples’ nonparametric Mann–Whitney U test to check that the imputed results were in line with the original data. Statistically significant difference was not observed (significance level criterion being 0.05; CI level, 95%) [23].

The background variables were not imputed. The subcategories of background variables having ≤ 5 answers were merged with another subcategory for statistical and identity-protection reasons (e.g., numbers of clinical teachers were combined with other clinical specialists for reporting). Likert scale categories “weekly” (= 5), “daily” (= 6) and “in every shift” (= 7) were merged forming only one category: “at least weekly” for the analysis. This was done due to the low numbers in category “daily” and no indications in category “in every shift”.

#### Exploratory factor analysis

Exploratory factor analysis [EFA] was used to test the construct validity and Cronbach’s alpha coefficient for measuring internal consistency. Explorative factor analysis is recommended for revealing latent structures of a phenomenon [31–33]. In the current study, EFA was used to reveal communication phenomena related to medication incidents. Aiming to find variable structures that could be generalized, were chosen the maximum likelihood method (ML) [32] for factor analysis. The approach was also used to assess the construct validity of the preliminary MIComHos-S1 and to reduce the excessive number of items used from the original literature-based MIComHos framework [32, 34].

**Fig. 2:**
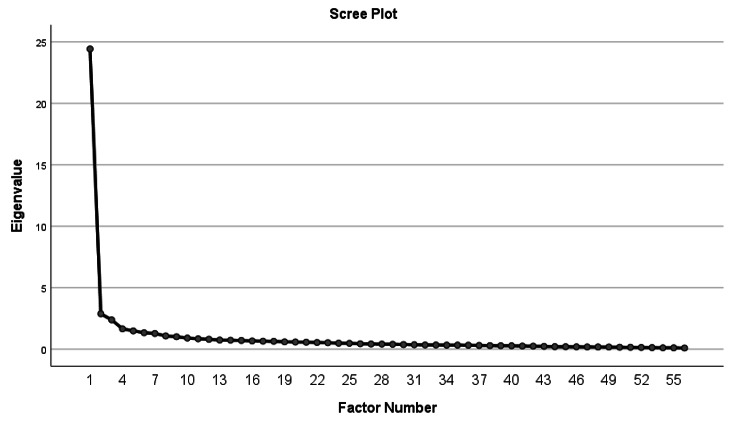
Scree plot for EFA. The scree plot is based on seven step Likert scale data describing health professionals’ perceptions of frequency of communication items contributing to medication incidents in hospitals

The factor number was determined using several methods. Kaiser’s eigenvalue > 1 provided 22 factors, and a bend in the scree plot graph suggested three to ten factors (Fig. 2) [32]. The use of the scree plot was based on a sample size greater than 100 (*n* = 303) [32], where Kaiser’s criterion is said to give high number of factors [32, 34, 35]. Due to major discrepancies in suggested factor numbers between Kaiser’s criteria and the scree plot suggestions, the factor number was finally decided using clarity-of-factor loadings and communalities. We aimed for loadings preferably greater than 0.400 (for n = 303) [32] and to find factor solution having least cross loadings between the factors and highest possible Cronbach’s alpha.

EFA resulted six-factor solution when the ML with oblique Promax rotation was used (Table 1). It was used because it allows factors to be correlated while also providing simpler, more interpretable factor structures (32). The factor correlation matrix was checked to confirm that all factors correlated between each other (0.297–0.698). The items were discarded one by one based on the lowest communality value until the lowest communality of item was above the cut off criteria of ≥ 0.30. The highest factor loading solution defined the items belonging to each factor and described the construct validity of the scale. The Cronbach’s alpha was used as a reliability measure to describe the internal consistency.

**Table 1:** Factoring solution and reliability evaluation of the MIComHos-S1 scale^†^

Factor number and name followed by the included items of the factor	Factor loading						Eigen value	Cumulative % of explained variance
Item number and name in the MIComHos-S1 scale	Factor 1	Factor 2	Factor 3	Factor 4	Factor 5	Factor 6		
**FACTOR 1: Communication regarding medication prescriptions (20 items; Cronbach´s alpha 0.949)**							43,367	43.367
65. Patient transfer between units or organisations was the situation of communication challenge	**0.89**	-0.235	0.097	-0.012	0.038	0.071		
70. Digital communication challenges contributed to medication incidents	**0.874**	-0.022	-0.08	-0.091	0.116	-0.091		
67. Incomplete or false information was transmitted between organisations	**0.76**	-0.073	0.074	0.031		0.049		
79. Transferring documentation between documents or systems caused information communication challenges	**0.707**	0.181		-0.129		-0.049		
66. Reporting handover during shift takeover was the situation of communication challenges	**0.644**	0.057	-0.068	0.175	0.027	0.032		
76. Unclear documentation system for medication dose	**0.618**	0.169	-0.103	0.011	0.038	0.033		
69. Oral communication challenges contributed to medication incidents	**0.566**	0.188	-0.117	0.22	0.083	-0.074		
26. Communication challenging with another person outside of own unit, but in the same organisation	**0.551**	-0.191	0.212	0.269	-0.098			
90. Was not aware that a new medication prescription was submitted outside of the routine ward round	**0.527**	**0.321**	0.049	-0.1	-0.034	-0.077		
24. Specialised healthcare unit - Specialised healthcare unit pair had challenges in communication	**0.515**	-0.231	0.279	0.296	-0.039	-0.016		
71. Communication over the phone contributed to medication incidents	**0.46**	0.28	0.025	0.132	0.1	-0.157		
58. Time pressure caused challenges for communication	**0.438**	0.062	0.063	0.086	0.123	0.076		
74. Printout copy of medication chart	**0.437**	0.189	-0.034	0.193	-0.158	0.055		
57. An error is repeated regularly, and all parties are aware of the challenge, but it has not been solved.	**0.393**	0.045	-0.199	0.207	0.086	0.148		
83. Colleague had false assumptions of someone’s factual actions	**0.384**	**0.342**		0.011	0.039	0.068		
14. Nurse-physician pair had challenges in communication	**0.383**	0.035	0.233	0.256	-0.195	0.043		
40. Incomplete, missing or unclear guidance along with medication prescription	**0.367**	0.229	-0.041	0.265		-0.029		
61. Disruption while dispensing/administering medication	**0.351**	0.145	0.095	-0.136	0.221	0.148		
49. Digital software restricted information retrieval	**0.338**	0.212	0.216	-0.076		-0.117		
73. Memo note, manual amendment into a printed medication chart, handwritten medication chart or folder	**0.332**	**0.306**	-0.159	0.204	-0.022	0.118		
**FACTOR 2: Communication regarding guidelines and reporting (15 items; Cronbach´s alpha 0.933)**							5,073	48.439
92. Guidance was not given about the issues that are to be observed due to the prescribed medication	0.113	**0.808**	0.047	-0.058	-0.052	-0.059		
89. Abbreviations or slang language (not standardised language)		**0.753**	0.032	-0.102	-0.109	0.126		
82. Documentation was lacking because the responsible person for documenting was not named for the ward round	0.041	**0.732**	0.024	-0.262	-0.097	0.149		
94. Reporting was lacking in case prescription was not implemented, an error occurred when implemented or the prescription was amended while implemented	-0.035	**0.668**	0.092	0.106	0.016	-0.018		
93. Effect of the medicine for the patient was not reported	-0.027	**0.658**	0.124	0.107	-0.03	-0.032		
50. Guidance for an exceptional situation was lacking	-0.238	**0.655**	0.165	0.045	0.029	-0.014		
88. Prescription was missing some information that would have been needed for implementation	**0.335**	**0.646**	0.015	-0.196	-0.077	0.057		
91. Mistake in interpretation of a prescription	0.182	**0.627**	-0.035	0.069		-0.011		
86. Incomplete or false documentation of oral prescription	0.249	**0.612**	0.025	0.019	-0.164	-0.034		
56. Guidance or advice not available for medication care	-0.168	**0.584**	0.015	0.141	0.041	0.062		
53. Equipment lacking for medication care	-0.099	**0.58**	0.103	0.052	0.023	0.061		
68. Not managed to contact a physician	0.285	**0.568**	-0.059	-0.089	0.107	-0.055		
55. Not aware of the guidance regarding medication care	-0.04	**0.507**	-0.035	0.29	0.036			
48. Regulation was restricting information retrieval or transmission	0.163	**0.492**	0.12	-0.092	0.027	-0.153		
54. Guidance or rule concerning medication care was lacking or was unclear	-0.089	**0.463**	0.03	0.287		0.077		
**FACTOR 3: Communication regarding patient and family member (9 items; Cronbach´s alpha 0.922)**							4,213	52.653
30. Patient knowingly did not tell about medication	-0.166	0.216	**0.749**	0.016	-0.057	-0.069		
28. Diverse cultural background of the patient	-0.062	0.01	**0.738**	-0.119	0.241	0.046		
27. Patient lacking language skills			**0.737**	-0.17	0.229			
29. Patient lacking mental abilities	-0.025	0.055	**0.698**	0.04	0.122	0.033		
31. Patient accidentally did not tell about medication	0.196	0.062	**0.682**	-0.049	-0.118	0.032		
33. Family member had incomplete or false information about the patient’s medication	0.131	0.032	**0.6**	0.118	0.067	-0.071		
32. Diverse professional groups had given confusing information to the patient about the medication	0.1	0.115	**0.527**	0.167	-0.031			
15. Nurse-patient pair had challenges in communication	0.159	-0.035	**0.494**	0.245	-0.113	0.09		
34. Medication was not discussed with the patient	0.166	0.114	**0.452**	0.173	-0.039	-0.024		
**FACTOR 4: Communication regarding implementation of guidelines (6 items; Cronbach´s alpha 0.877)**							2,964	55.617
41. Professional did not seek advice regardless of feeling unconfident	0.051	0.13	-0.101	**0.726**	0.15	-0.037		
13. Nurse-nurse pair had challenges in communication	0.262	-0.176	0.017	**0.724**	-0.111	-0.025		
25. Within the home unit among the colleagues were challenges in communication	0.228	-0.166	0.113	**0.693**	-0.108	0.02		
42. Not aware of the existing rule or guidance concerning medication care	-0.15	**0.326**		**0.621**	0.015	0.024		
38. Professionals aware of guidance given by the organisation, but the rules were bent	0.263	-0.066		**0.465**	0.086	-0.025		
39. Personal characteristics of colleague challenged communication about medication	-0.034	0.26	0.045	**0.428**	0.158	0.031		
**FACTOR 5: Communication about competencies and responsibilities (4 items; Cronbach´s alpha 0.828)**							2,61	58.227
43. Lacking language skills contributed to probability of medication error	0.058	-0.148	0.109		**0.862**	0.041		
45. Diverse cultural background challenged communication	0.014	0.025	0.139	-0.075	**0.801**	-0.026		
46. Within the home unit, it was unclear who was the responsible person for the medication	0.051	0.207	-0.175	0.298	**0.382**			
44. The competence required in the clinical unit was not met by the temporary staff	0.019	0.22	0.087	0.144	**0.377**	0.059		
**FACTOR 6: Communication regarding attitude and atmosphere (3 items; Cronbach´s alpha 0.843)**							2,516	60.743
63. Medication incident was not reported because the atmosphere in the clinic is not encouraging to do so		0.085	-0.024		-0.092	**0.956**		
64. Fear of authorities or getting humiliated is challenging communication	-0.136	0.128	-0.026	0.07	0.1	**0.637**		
62. Medication errors are not reported because the previous reports have not generated any actions	0.181	0.035	0.057	-0.093	0.097	**0.63**		
VALUES FOR THE ENTIRE SCALE **(57 items; Cronbach´s alpha 0.976)**								
Extraction Method: Maximum Likelihood.								
Rotation Method: Promax with Kaiser Normalization.								
a Rotation converged in 10 iterations.								
MIComHos-S = Medication Incidents and Communication in Hospital-scale								

^†^ MIComHos-S1 = Medication Incidents and Communication in Hospitals -Scale

#### Analysis of communication challenge frequencies

The number of responses in each frequency category in Likert scale (e.g., monthly, weekly or daily) were analysed for each item (= challenge or communication promoting issue). The items having the highest number of respondents perceiving the detailed challenge “at least weekly” (= Merged Likert scale category 5 = merged categories of 5 [weekly], 6 [daily] and 7 [every working shift]) are reported in this paper.

## Results

The study produced the preliminary MIComHos-S1 comprising six communication factors covering 57 items altogether. The scale was developed for measuring the health professionals’ perceptions of frequencies of communication challenges or promoting issues contributing to medication incidents in hospitals. The scale’s preliminary construct validity and internal consistency were acceptable.

### Sample characteristic

The final sample consisted of n = 303 respondents. More than three quarters of the respondents were registered nurses, but also physicians, pharmacists, specialists and practical nurses were represented. Over half of respondents worked in inpatient departments (*n* = 170; 56.1%), but wide variety of department types were represented from outpatient clinics to intensive care units and operating theatres. Hierarchal positions from frontline staff to chief positions were represented, managers and chiefs covering fifth of the respondents. Nearly half of respondents had five years or less (n = 131; 43.2%) work experience in current position. Majority of respondents had submitted incident reports by themselves and had ward pharmacist available in the unit. (Table 2.)

**Table 2:** Sample characteristic (*n* = 303)

Background variables	n	%	Number (%) of missing responses	Total
**Location of respondents’ work unit (reclassified due to < 5 respondents/category)**	n = 303		0(0)	303
In hospital	288	95		
Outpatient service within hospital, or service provided off the hospital site, or having several locations	15	5		
**Type of work unit**	n = 303		0(0)	303
Inpatient department	170	56.1		
Outpatient clinic, day surgery	50	16.5		
Intensive care unit, operating theatre, anesthesia unit	49	16.2		
Something else	10	3.3		
Several working units/areas	24	7.9		
**Hierarchy position (reclassified)**	n = 301		2(0.7)	303
Not in management position	240	79.2		
Immediate management position	49	16.2		
Middle management or chief position	12	4.0		
**Professional group**	n = 297		6(2.0)	303
Practical nurse	12	4		
Registered nurse	235	77.6		
Physician or specialized physician	25	8.3		
Pharmacist	15	5.0		
Specialist (nursing/medical/patient safety/clinical teacher (medical or nursing)	10	3.3		
**Work experience in this organization in current position (reclassified)**	n = 283		20(6.6)	303
0–5 years	131	43.2		
6–15 years	99	32.7		
16 years or more	53	17.5		
**Work experience in current type position altogether (reclassified)**	n = 289		14(4.6)	303
0–5 years	84	27.7		
6–15 years	122	40.3		
16 years or more	83	27.4		
**Availability of clinical pharmacist services in the clinical area of the responder (reclassified)**	n = 295		8(2.6)	303
Not available or not sure	60	19.8		
Yes available	235	77.6		
**Submitted a digital incident report himself/herself concerning medication error**	n = 300		3(1.0)	303
Not submitted	38	12.5		
Yes submitted	262	87.5		
**Percentage of factual medication incidents that are entered into a digital incident register (perception of respondent)**	n = 277		26(8.6)	303
0–20%	50	16.5		
30–40%	67	22.1		
50–60%	99	32.7		
70–80%	54	17.8		
90–100%	7	2.3		
**Regularity of analyzing incident reports with staff by manager or patient safety specialist**	n = 300		3(1.0)	303
Every day	8	2.6		
Weekly	61	20.1		
Monthly	117	38.6		
Few times per year or once a year	106	35.0		
Never analyzed together	8	2.6		
**Perception of getting sufficient information concerning the developments generated based on the incident reports**	n = 296		7(2.3)	303
Not sufficient	124	40.9		
Yes sufficient	167	55.1		
It is not my responsibility area	5	1.7		
**Years the current digital medication management system has been in use in the clinical area**	n = 287		16(5.3)	303
Does not know	68	22.4		
Around one year or less	31	10.2		
Several years	171	56.4		
Old and new systems are overlapping currently. We are shifting to the new system	17	5.6		

### Construct validity

In the final EFA, the sampling was adequate (Kaiser–Meyer–Olkin, 0.954) being > 0.5 [32], factoring method appropriate for these items (Bartlett’s Test of Sphericity, *p* < 0.001) and measured goodness-of-fit test statistically significant (chi-square, *p* < 0.001) concerning communalities before and after item discard [32]. Table 1 presents the acceptable values of variance and eigenvalues per factor.

The six factors solution gave the clearest item groups, with the highest item loadings to factors (0.332–0.890) and the lowest number of cross loadings (five slight cross loadings) between the factors. The first factor was about communication related to medication prescriptions, which explained the variance of 43.367 between the factors. All six factors explained the variance of 60.743%, with the lowest eigenvalue being 2.516 when the minimum requirement for the eigenvalue is > 1 [32]. Factor loadings of the six factors are presented in Table 1. Altogether, 57 items were retained of the original 82 items. The lowest communality of the retained items was 0.329.

### Factor construct

#### Factor 1 - communication regarding medication prescriptions

Twenty items were loaded for factor 1, “Communication regarding medication prescriptions.” The item loadings ranged between 0.332 and 0.89. Three items of the medication prescription–related factors slightly cross-loaded to factor 2. The items included the following: “Not aware about prescriptions given outside of normal ward round” (0.321); “Colleagues had false assumptions about colleagues’ actions” (0.342) (= actions not communicated); and an item concerning memo notes, manual adds to a printed document and manual medication lists or folders (0.306) (= challenge relating to a communication method). The cross loadings indicated linking with communication about guidelines and reporting.

#### Factor 2 - communication regarding guidelines and reporting

Fifteen items were loaded for factor 2, “Communication regarding guidelines and reporting.” Loadings ranged between 0.463 and 0.808. The cross loading was for the factor of medication prescription, concerned the following item: “There were instructions lacking in medication prescription” (0.335). This is in line with the reality that factors 1 and 2 are closely linked regarding the item.

#### Factor 3 - communication regarding patient and family member

Nine items were loaded for factor 3, “Communication regarding patient and family member,” with items loading between 0.452 and 0.749. There were no cross-loadings regarding communication with patients and family.

#### Factor 4 - communication regarding implementation of guidelines

Six items were loaded for factor 4, “Communication regarding implementation of guidelines, having items loading between .428–.726.” One item from the factor regarding guidelines and reporting: “Not aware of the existing rule or guidance concerning medication care” (0.326) (= lack of communication about guidelines) cross-loaded with the factor for guideline implementation. This supports the solution for these two separate factors regarding “Communication about guidelines” and “Communication about the implementation of guidelines.”

#### Factor 5 - communication about competencies and responsibilities

Four items were loaded for factor 5, “Communication about competencies and responsibilities,” with loading rates ranging from 0.377 to 0.862. No cross-loadings were extracted.

#### Factor 6 - communication regarding attitude and atmosphere

Three items were loaded for factor 6, “Communication regarding attitude and atmosphere,” with items loading from 0.63 to 0.956. No cross-loadings were extracted in this factor.

### Internal consistency

The internal consistency values for all factors (Cronbach’s *α* coefficients between 0.828 and 0.949) and the entire scale (*α* = 0.976) were acceptable [32, 36]. Cronbach’s α values of all factors suggested retaining all remaining items. (Table 3.)

**Table 3:** The factors of MIComHos-S1 with internal consistency values

Factor number	Factor name	Number of items	Cronbach’s alpha †
Factor 1	Medication prescription	20	0.949
Factor 2	Guidelines and reporting	15	0.933
Factor 3	Patients and family members	9	0.922
Factor 4	Implementation of guidelines	6	0.877
Factor 5	Competencies and responsibilities	4	0.828
Factor 6	Attitude and atmosphere	3	0.843
**Total**	**MIComHos-S1**	**57 items**	**0.976**

† Cronbach’s alpha (α,) is a value of internal consistency of factors and the scale, which are preferred to be above α, 0.70 (Field, 2018, p.823; Greco et al., 2018)

### The most common weekly perceived communication challenges

**Fig. 3:**
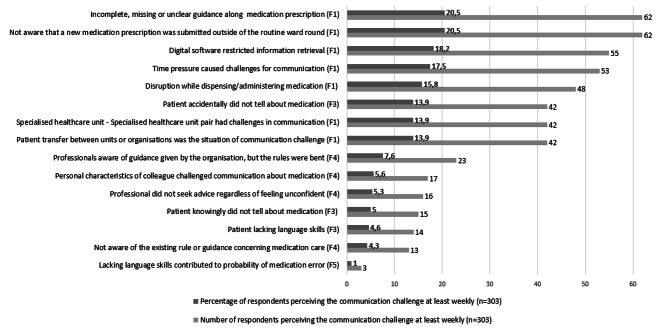
Likert scale result examples when using MIComHos_S1. Examples of “at least weekly” occurring communication challenges, which contribute to medication incidents in hospitals, perceived by health professionals in specialized healthcare (n = 303). * Measured with MIComHos-S1 (= Medication Incidents and Communication in Hospitals-Scale, version one)

The most common weekly communication challenges concerned medication “prescription related factor” (Factor 1), and secondly “patient and family related factor” (Factor 3) (Fig. 3). The results suggested that the most usual, “at least weekly” perceived challenge were lack of communication about prescriptions or prescriptions being incomplete or unclear (n = 62;20,5%). A specific weekly communication challenge was missing prescriptions when they were written digitally outside of the regular physicians’ ward-rounds (n = 62;20,5%) (= missed communication). In addition, digital software was perceived restricting medication information retrieval weekly (n = 55;18,2%). Also, time pressure (n = 53;17,5%) and disruptions (n = 48;15,8%) challenged medication communication at least weekly. Regarding the “Patient and family” factor (Factor 2) professionals’ perceptions revealed that patients do not talk about their medication accidentally (n = 42;13,9%) (= lack of communication). Furthermore, professionals perceived weekly that patients also knowingly did not talk about their medication (n = 15; 5%) (= lack of communication) and that patients’ language skills challenge communication (n = 14;4,6%). Instead, fewer professionals perceived staffs’ language skills contributing to medication incidents (n = 3;1%). (Fig. 3.)

Overall, the most frequent communication challenges were perceived among the respondents who were responsible for more than one unit or clinical area. The respondents acting in high hierarchy positions experienced more frequent challenges in guideline implementation communication and communication regarding atmosphere than respondents in lower positions.

## Discussion

The study modified the “Medication Incidents and Communication in Hospital” framework and reduced its items into a preliminary MIComHos-S1 scale for measuring health professionals’ perceptions about frequencies of communication items contributing to medication incidents. The construct validity and internal consistency of the MIComHos-S1 were acceptable. The scale development was a response to the appeal in previous scientific literature [21, 22]. The scale was developed in accordance with Steiner’s and Knotter’s [37] scale-development phases (excluding confirmatory factor analysis validation), which strengthened the credibility of the scale. The MIComHos-S1 is grounded to solid evidence-base [38, 39] by combining scientific literature evidence, the expertise of clinical healthcare practitioners and patient representatives.

### The scale

The literature-based communication items were reduced by one-fifth with the help of expert panellists, which increases the usability of the scale. The factor number and distribution of items within the factors after EFA were distinct to the main categories of the original MIComHos framework and the concept [11, 22]. Communication dyads, instead of forming their own factor, some of dyads were relocated within the other factors, suggesting the dyads are meaningful only as part of specific factors. The solution is in line with contemporary patient safety ideology, in which the meaning of system failure is emphasized instead of personal failure [1, 40].

The category of “Medication prescription–related issues” of the MIComHos framework was extracted as own factor also in the MIComHos-S1. However, items regarding “Institutional issues” and “Structural and process issues” were organized differently by EFA than those arranged in the literature based MIComHos framework. Interestingly, separate factors were extracted for communication challenges concerning “Guidelines and reporting” and “Implementation of guidelines.” This result highlights the fact that just informing professionals about guidelines does not lead to guideline implementation – also communication about implementation is needed. The factor solution is supported by the previous studies [41, 42], which reported healthcare professionals knowingly bending medication safety guidance. Similarly, there is evidence in literature about the culture of the “normalization of deviation” regarding medication safety guidelines [43]. Thus, reflecting the findings in the previous studies, the factor solution suggests that specific communication about implementation actions is meaningful.

Communication related to patients and families was extracted as its own factor. This mirrored the numerous findings regarding the pivotal role of communication with patients and families about medication and medication care [4, 44, 45]. Armitage and colleagues [46] concluded in their exploratory comparative study of patient safety data that some patient safety incidents may not even be found in any other realm than by communicating with patients. This supports the need for a specific factor of communication with patients and families.

Communication attitudes and atmosphere items were extracted as their own factor; hence it looks like those might represent important underlying phenomena for medication safety. The results support the findings reported by the studies measuring patient safety culture in hospitals. Open non-punitive patient safety culture opens the possibility for organizations to learn from mistakes [19, 47], and as such, communication attitudes and atmosphere appear related to medication incidents.

### Construct validity and internal consistency of the scale

The construct validity of the scale was acceptable. The recommended criterion, by Costello and Osborne [35], of a minimum of five items loading at a minimum of 0.50 was achieved for half of the six factors. The communalities for single items were expected to be greater than 0.32–0.60 [32, 35], of which the lowest was achieved. Internal consistency of the entire scale was excellent (α = 0.976) being way above 0.70. The scale included 57 items, which might have increased Cronbach’s alpha (α = 0.828–0.949) causing bias to the results [23, 37, 48].

The final scale had five slight cross loading items, which each were placed in the highest loading factor. The reasons for cross loadings were clearly recognizable. However, it was important still to keep both factors separately. For example, the item “Not aware of the existing rule or guidance concerning medication care” loaded strongest to the factor “Guideline implementation” but had slight cross loading with the factor “Communication about guidelines”. Retaining both factors was important, as informing about guidelines is necessary, but it is not enough. Also, guideline implementation needs to be prompted via administrative communication to secure factual implementation of guidelines in practice.

### Weekly communication challenges

Use of the MIComHos-S1-scale produced information of communication challenges both on detailed and phenomena level, which can be used in clinical front-line intervention planning and administrative development.

According to the factor level results the highest factor levels were for the highest position professionals. It might come up due to their condensed view of incident reports in wide clinical area. However, this is not necessarily causing bias in the results as the respondents were asked their perceptions regarding their responsibility area. Overall, factor levels deflate the Likert results but mirror accurately the most frequent “at least weekly” perceived communication challenges.

### Study recommendations

To complete the scale-development process, a confirmatory factor analysis (CFA) is needed with a new sample to validate the MIComHos-S1 [31, 37, 49]. The preferred sample size should be ten times larger than the number of items in the scale (> 570) [37]. Test–retest or split-half parallel analysis and assessment of difference of the CFA results from two groups should be used for reliability analysis of the scale in the future [50]. After validation, the scale could be used to assess the most frequent communication factors, which could help to prioritize where to apply interventions to improve medication safety in hospital. The Likert scale results from item level could be used for assessing specific challenges.

The MIComHos-S1 scale was developed for annual or biannual use in healthcare units or organizations for assessing the existence of main communication challenges contributing to medication incidents. The data could be collected and analysed for example by quality managers, directors or managers. Alternatively, the scale questions can be used as reflection framework in team meetings to discuss the communication challenges contributing to medication incidents. The scale is not suitable for daily use due to its length, nor measuring the communication challenges even monthly is necessary, as solving communication challenges may require lengthy organizational level changes.

The scale is transferable between countries and healthcare settings. The full potential of the scale is achieved if the healthcare setting is using electronic health records, incident reporting systems and where medication prescriptions are written and used. As the scale measures frequency perceptions for each communication challenge type, it is possible to answer “never” if the challenge type is not applicable in the own setting.

### Limitations and strengths

The results of this study are limited to healthcare professionals’ perceptions of the communication challenges related to medication incidents in specialized healthcare in Finland.

Face validity of the preliminary scale was assessed using expert panellists [51]. To maximize the sample heterogeneity, the participating units were selected in cooperation with the contact person of the organization, resulting in potential bias. One large clinic preferred not to contribute to the study but did not provide a reason for this decision, which increased the risk of sample bias.

The response rate to digital survey was low (8.84% n = 344, of n = 3,892 eligible participants) regardless of a strong effort in the recruitment process. The length of the questionnaire might have limited the number of respondents [26]. Low response rates in surveys can be seen quite often in resent studies among healthcare professionals [26]. Clinical work nowadays is hectic, and the workload is high due to staff shortages. This limits the time available for additional duties like answering research questionnaires. Therefore, long questionnaires are prone to remain uncompleted even if answering was started. This might be a reason for the fact that 41 statistical units in this study had to be discarded due to missing values criteria; thus, n = 303 was used as the final sample. This might have caused bias in the results, but the discarded statistical units did not represent any specific group. Thus, the results are cautiously indicative of the populations included in the study organizations.

Although the final sample size (n = 303) was low, it represented all unit types, professional groups and hierarchy levels of targeted groups, mirroring the trend of the group ratio. Although the number of respondents did not fulfil the strictest qualifications for the number of respondents for factor analysis [35, 49], the number met the minimum ratio of 1:2 [30, 49] and the criteria outlined by [52], requiring a minimum of 250 respondents and three to six observations per item in the analysis. In addition, the number satisfied the rule of thumb of having a minimum of 300 respondents for factor analysis [32, 49].

Using MI caused a risk of bias in the Likert-scale values [28]. However, the increase in total bias due to imputation was not regarded as significant based on the similarity of the results between the original and imputed data. The final number of items in the preliminary scale was reduced by the current EFA, which might increase the response rate in the future.

The scale development of the MIComHos-S1 is in its infancy, which can be recognize as five cross loadings in the scale. Also, the item number for the factor of “attitude and atmosphere” is low. The factor might need strengthening with additional items in the future to get the scale more balanced. The fact that the items of communication dyads were spread across the other factors during factor analysis is suggesting that the dyad items might be reduced from the scale in the future.

Guidelines for reporting observational studies (STROBE) checklist was used in reporting the study.

## Conclusions

The preliminary MIComHos-S1 scale of six communication factors covering 57 communication items achieved acceptable construct validity and internal consistency. Confirmatory factor analysis is needed for the scale validation. The scale looks promising for describing the main communication factors contributing to medication incidents, but the scale is in its infancy. After validation, the scale can be used for determining detailed communication challenges to direct intervention to the most frequent communication challenges. The weekly perceived communication challenges suggest that interventions are needed to standardize prescribing documentation and to strengthen communication about prescriptions given outside of regular ward-rounds.

### Electronic supplementary material

Below is the link to the electronic supplementary material.

Supplementary Material 1

## Data Availability

The data that support the findings of this study are available from the corresponding author upon reasonable request.

## Abbreviations

CFA: Confirmatory Factor Analysis
CI: Confidence Interval
CVI: Content Validity Index
CVR: Content Validity Ratio
DF: Degree of Freedom
EFA: Exploratory Factor Analysis
MCAR: Missing Completely at Random
PMM: Predictive Mean Matching Method
MI: Multiple Imputation
MIComHos: Medication Incidents and Communication in Hospitals
MIComHos-S1: Medication Incidents and Communication in Hospitals –Scale, version 1
ML: Maximum Likelihood Method
SPSS: Statistical Package for Social Sciences
US: United States
WHO: World Health Organization
α: Cronbach’s alpha
