# Supplementary material for: Measuring health professionals’ perceptions of communication contributing to medication incidents in hospitals - scale development and primary results of weekly perceived communication challenges
Source: BMC Nurs. 2023 Aug 25;22:285. doi: 10.1186/s12912-023-01455-x (PMC10463788; doi:10.1186/s12912-023-01455-x)
Supplement: Supplementary file 1 — Supplementary Material 1 [file 12912_2023_1455_MOESM1_ESM.docx]

**SUPPLEMENTARY TABLE 1** Factor levels within a background variable subgroup and statistical differences of factor’s levels between subgroups (*n*=303)

|  | **Factor 1 mean** | **Factor 2 mean** | **Factor 3 mean** | **Factor 4 mean** | **Factor 5 mean** | **Factor 6 mean** | **Mean value of the factor levels of the variable** | **Mean SD range of factor levels within the subgroup:** |
| --- | --- | --- | --- | --- | --- | --- | --- | --- |
| **Factor mean (SD), min; max (n=303)** | 2.99 (0.88), 1.55; 5.51 | 2.23 (0.77), 1.00; 5.23 | **3.03** (0.90), 1.00;  5.56 | 2.83 (0.89),  1.00;  5.33 | **1.97** (0.81),  1.00;  5.50 | 2.14 (1.13),  1.00;  6.0 | 2.53 | - |
| **Background variables followed by the variable’s subgroups:** |  |  |  |  |  |  |  |  |
| **1. Location of the clinical unit (*n* = 303)** |  |  |  |  |  |  |  |  |
| Hospital inpatient unit (*n* = 288) | 3 | 2.23 | **3.04** | 2.82 | ***1.97*** | 2.11 | *2.53* | 0.77-1.09 |
| Outpatient service (in hospital or outside the hospital) or responsible for several units (*n =* 15) | 2.94 | 2.43 | 2.97 | **3.22** | ***2.07*** | 2.81 | 2.74 | 0.66-1.59 |
| *P*-value of the difference in mean values between the subgroups within one factor | *†*   *.795* | *† .445* | *† .996* | *† .143* | *† .357* | *† .082* |  |  |
|  |  |  |  |  |  |  |  |  |
| **2. Type of the clinical unit (*n* = 303)** |  |  |  |  |  |  |  |  |
| Inpatient unit (*n =* 170) | 3.1 | 2.3 | ***3.13*** | 2.84 | ***2.12*** | 2.16 | 2.61 | 0.75-1.13 |
| Outpatient clinic, day surgery or imaging (*n =* 50) | 2.42 | 1.94 | ***2.85*** | 2.36 | ***1.62*** | 1.75 | *2.16* | 0.63-1.04 |
| Intensive care unit, operating theatre, anaesthesia unit (*n =* 49) | ***2.85*** | 2.04 | 2.62 | 2.82 | ***1.71*** | 2.02 | 2.34 | 0.74-1.01 |
| Something else (*n =* 10) | ***3.71*** | 2.86 | 3.44 | 3.65 | ***2.07*** | 3.31 | 3.17 | 0.77-1.27 |
| Responsible for several units (*n =* 24) | 3.46 | 2.6 | 3.37 | ***3.51*** | ***2.16*** | 2.66 | 2.96 | 0.54-1.02 |
| *P*-value of the difference in mean values between the subgroups within one factor | ‡ .000  *** | ‡ .000  *** | ‡ .001  *** | ‡ .000  *** | ‡ .000  *** | ‡ .000  *** |  |  |
|  |  |  |  |  |  |  |  |  |
| **3. Position (*n* = 301)** |  |  |  |  |  |  |  |  |
| Not in managerial position (*n* = 240) | 2.95 | 2.23 | ***3.04*** | 2.79 | ***1.97*** | 2.2 | 2.53 | 0.77-1.13 |
| Immediate manager (*n* = 49) | ***3.08*** | 2.17 | 2.93 | 3.01 | 1.92 | ***1.82*** | *2.49* | 0.72-1.04 |
| Middle management or lead position (*n =* 12) | 3.75 | 2.74 | 3.4 | ***3.44*** | ***2.18*** | 2.58 | 3.02 | 0.83-1.25 |
| *P*-value of the difference in mean values between the subgroups within one factor | ‡ .053 | ‡ .090 | ‡ .398 | ‡ .026  * | ‡ .615 | ‡ .019  * |  |  |
|  |  |  |  |  |  |  |  |  |
| **4. Professional Group (*n* = 297)** |  |  |  |  |  |  |  |  |
| Practical nurse (*n =* 12) | 2.45 | 2.1 | ***2.68*** | 2.48 | ***1.88*** | 2.32 | *2.32* | 0.79-1.43 |
| Registered nurse (*n =* 235) | 2.93 | 2.17 | ***2.99*** | 2.76 | ***1.96*** | 2.08 | 2.48 | 0.73-1.07 |
| Physician or specialised physician (*n =* 25) | ***3.3*** | 2.49 | 3.16 | 3.12 | ***1.88*** | 2.03 | 2.66 | 0.72-1.03 |
| Pharmacist (*n =* 15) | ***3.68*** | 2.8 | 3.48 | 3.52 | ***2.11*** | 2.8 | 3.07 | 0.72-1.26 |
| Clinical nurse specialist, clinical educator (nursing/medicine), patient safety specialist or something else (*n =* 10) | 3 | 2.25 | ***3.27*** | 2.96 | ***2.17*** | 2.28 | 2.66 | 0.85-1.60 |
| *P*-value of the difference in mean values between the subgroups within one factor | ‡ .007  ** | ‡ .019  * | § .116 | ‡ .008  ** | ‡ .651 | ‡ .217 |  |  |
|  |  |  |  |  |  |  |  |  |
| **5. Clinical pharmacist available in the clinical setting (*n* = 295)** |  |  |  |  |  |  |  |  |
| No or I do not know (*n* = 60) | 2.51 | 2.06 | 2.59 | ***2.61*** | ***1.73*** | 1.92 | *2.24* | 0.71-1.17 |
| Yes (*n* = 235) | 3.12 | 2.28 | ***3.16*** | 2.9 | ***2.03*** | 2.19 | 2.61 | 0.75-1.09 |
| *P*-value of the difference in mean values between the subgroups within one factor | † .000  *** | † .033  * | † .000  *** | † .035  * | † .007  ** | † .028  * |  |  |
|  |  |  |  |  |  |  |  |  |
| **Submitted a medication incident report by herself/himself (*n =* 300)** |  |  |  |  |  |  |  |  |
| no (*n* = 38) | 2.75 | 2.22 | ***2.88*** | 2.64 | ***1.78*** | 2.01 | *2.38* | 0.77-1.27 |
| yes (*n* = 262) | 3.03 | 2,24 | ***3,06*** | 2,87 | ***2*** | 2,18 | 2,56 | 0.76-1.11 |
| *P*-value of the difference in mean values between the subgroups within one factor | † .053 | † .699 | † .390 | † .136 | † .068 | † .150 |  |  |
|  |  |  |  |  |  |  |  |  |
| **6. Percentage of the real medication incidents that are eventually reported into the digital system in the clinical setting (*n* = 277)** |  |  |  |  |  |  |  |  |
| 0-20% (*n =* 50) | 3.28 | 2.58 | ***3.34*** | 3.04 | ***2.05*** | 2.61 | 2.82 | 0.88-1.49 |
| 30-40% (*n =* 67) | ***3.33*** | 2.51 | 3.2 | 3.17 | ***2.16*** | 2.35 | 2.79 | 0.76-1.10 |
| 50-60% (*n =* 99) | 3.03 | 2.19 | ***3.09*** | 2.89 | ***1.98*** | 2.22 | 2.57 | 0.73-0.99 |
| 70-80% (*n =* 54) | 2.79 | 2.06 | ***2.92*** | 2.64 | ***1.88*** | 1.89 | 2.36 | 0.58-0.96 |
| 90-100% (*n =* 7) | ***1.85*** | 1.35 | 1.73 | 1.78 | 1.29 | ***1.21*** | *1.54* | 0.28-0.80 |
| *P*-value of the difference in mean values between the subgroups within one factor | ‡ .000  *** | ‡ .000  *** | ‡ .001  *** | ‡ .000  *** | ‡ .013  ** | ‡ .005  ** |  |  |
|  |  |  |  |  |  |  |  |  |
| **7. Analysis frequency of incident reports in the clinical unit with staff (*n* = 288)** |  |  |  |  |  |  |  |  |
| Every day (*n =* 8) | 2.86 | 2.11 | ***2.95*** | 2.59 | 1.84 | ***1.64*** | *2.33* | 0.60-1.19 |
| Weekly (*n =* 61) | 3.02 | 2.25 | ***3.11*** | 2.93 | ***2*** | 2.07 | 2.56 | 0.73-1.17 |
| Monthly (*n =* 117) | 2.94 | 2.17 | ***2.98*** | 2.79 | ***1.88*** | 2.01 | 2.46 | 0.68-1.03 |
| Few times per year (*n* = 82) and I do not know (*n =* 12) | 3 | 2.24 | ***3.03*** | 2.81 | ***2*** | 2.3 | 2.56 | 0.83-1.14 |
| Never analysed with staff (*n =* 8) | ***3.37*** | 3.01 | 3.34 | 3.14 | ***2.56*** | 3.11 | 3.09 | 0.84-1.73 |
| *P*-value of the difference in mean values between the subgroups within one factor | § .701 | ‡ .249 | §  .737 | ‡ .598 | ‡ .270 | ‡ .080 |  |  |
|  |  |  |  |  |  |  |  |  |
| **8. Satisfaction for amount of information regarding the generated actions based on incident reports (*n* = 296)** |  |  |  |  |  |  |  |  |
| Not satisfied (*n =* 124) | **3.16** | 2.38 | 3.15 | 3.03 | **2.1** | 2.51 | 2.72 | 0.81-1.25 |
| Yes satisfied (*n =* 167) | 2.87 | 2.12 | **2.96** | 2.7 | **1.85** | 1.88 | *2.40* | 0.70-0.93 |
| It is not in my area of responsibility (*n =* 5) | 3.28 | 2.39 | **3.33** | 3.23 | 2.63 | **1.87** | 2.79 | 0.21-0.86 |
| *P*-value of the difference in mean values between the subgroups within one factor | ‡ .019  * | ‡ .021  * | ‡ .225 | ‡ .008  ** | ‡ .003  ** | ‡ .000  *** |  |  |
|  |  |  |  |  |  |  |  |  |
| **9. Age of the digital system in use for medication management in the clinical setting (*n* = 287)** |  |  |  |  |  |  |  |  |
| I do not know (*n =* 68) | 2.86 | 2.28 | **2.95** | 2.75 | **1.94** | 2.17 | 2.49 | 0.79-1.21 |
| One year or less (*n =* 31) | 2.97 | 2.2 | **3.14** | 2.61 | 2.04 | **1.81** | *2.46* | 0.81-1.01 |
| Several years (*n =* 171) | 3.05 | 2.24 | **3.07** | 2.94 | **1.97** | 2.24 | 2.59 | 0.75-1.11 |
| Old and new digital system overlapping currently - we are in transition phase (*n =* 17) | **3.24** | 2.35 | 3.02 | 2.81 | **1.9** | 1.91 | 2.54 | 0.45-1.20 |
| *P*-value of the difference in mean values between the subgroups within one factor | § .302 | ‡ .784 | ‡ .587 | ‡ .165 | ‡ .867 | ‡ .117 |  |  |
|  |  |  |  |  |  |  |  |  |
| **10. Work experience in current organisation in current position (*n* = 283)** |  |  |  |  |  |  |  |  |
| 0-5 years (*n* = 131) | 3.09 | 2.36 | **3.15** | 3.00 | **2.03** | 2.39 | 2.67 | 0.83-1.31 |
| 6-15 years (*n* = 99) | 2.97 | 2.11 | **2.99** | 2.73 | **1.92** | 2.02 | 2.46 | 0.70-0.93 |
| 16 years or more (*n* = 53) | 2.84 | 2.14 | **2.93** | 2.65 | 1.90 | **1.73** | *2.37* | 0.74-0.88 |
| *P*-value of the difference in mean values between the subgroups within one factor | ‡ .187 | ‡ .026  * | §  .230 | § .018  * | ‡ .474 | ‡ .003  ** |  |  |
|  |  |  |  |  |  |  |  |  |
| **11. Work experience in current position type altogether (*n* = 289)** |  |  |  |  |  |  |  |  |
| 0-5 years (*n* = 84) | 3.09 | 2.40 | **3.15** | 3.10 | **1.97** | 2.22 | 2.65 | 0.74-1.14 |
| 6-15 years (*n* = 122) | 2.94 | 2.16 | **2.97** | 2.78 | **2.02** | 2.21 | 2.51 | 0.79-1.15 |
| 16 years or more (*n* = 83) | 2.95 | 2.14 | **3.06** | 2.68 | 1.93 | **1.90** | *2.44* | 0.77-1.02 |
| *P*-value of the difference in mean values between the subgroups within one factor | ‡ .356 | ‡ .019  * | ‡ .456 | ‡ .009  ** | ‡ .720 | §  .097 |  |  |

Font style code: **Bolded** values of factor levels are the extremity values on the variable’s row

Abbreviations: **F1**: Communication regarding medication prescription , **F2**: Communicated guidelines and reports , **F3**: Communication with patient and family members  , **F4**: Communication about implementation of guidelines , **F5**: Communication about competencies and responsibilities  , **F6**: Communication attitude and atmosphere ; **Factor mean level range**: 1 = never, 2 = odd occasion during the last year, 3 = couple times per year, 4 = monthly, 5 = weekly, 6 = daily, 7 = in every working shift; **†** : Mann-Whitney U-test; **‡**: Kruskall Wallis test; **§**: One Way Anova test; *****:  P≤0.05; ****** : P≤ 0.01; ******* : P≤0.001
